# Sustained delivery of recombinant human bone morphogenetic protein-2 from perlecan domain I - functionalized electrospun poly (ε-caprolactone) scaffolds for bone regeneration

**DOI:** 10.1186/s40634-016-0057-1

**Published:** 2016-10-06

**Authors:** Yu-Chieh Chiu, Eliza L. Fong, Brian J. Grindel, Fred K. Kasper, Daniel A. Harrington, Mary C. Farach-Carson

**Affiliations:** 1 Fischell Department of Bioengineering, University of Maryland, 2212 Jeong H. Kim Building, College Park, MD 20742 USA; 2 Department of Biomedical Engineering, National University of Singapore, Singapore, Singapore; 3 Department of Bioengineering, Rice University, 6500 Main Street, Houston, TX 77030 USA; 4 Department of Orthodontics, The University of Texas Health Science Center at Houston, 7500 Cambridge St, Houston, TX 77054 USA; 5 Department of BioSciences, Rice University, 6500 Main Street, Houston, TX 77030 USA

**Keywords:** Heparan sulfate, Poly(ε-caprolactone), Bone morphogenetic protein, Alkaline phosphatase, Perlecan/HSPG2, Bone regeneration

## Abstract

**Background:**

Biomaterial scaffolds that deliver growth factors such as recombinant human bone morphogenetic proteins-2 (rhBMP-2) have improved clinical bone tissue engineering by enhancing bone tissue regeneration. This approach could be further improved if the controlled delivery of bioactive rhBMP-2 were sustained throughout the duration of osteogenesis from fibrous scaffolds that provide control over dose and bioactivity of rhBMP-2. In nature, heparan sulfate attached to core proteoglycans serves as the co-receptor that delivers growth factors to support tissue morphogenesis.

**Methods:**

To mimic this behavior, we conjugated heparan sulfate decorated recombinant domain I of perlecan/HSPG2 onto an electrospun poly(ε-caprolactone) (PCL) scaffold, hypothesizing that the heparan sulfate chains will enhance rhBMP-2 loading onto the scaffold and preserve delivered rhBMP-2 bioactivity.

**Results:**

In this study, we demonstrated that covalently conjugated perlecan domain I increased loading capacity of rhBMP-2 onto PCL scaffolds when compared to control unconjugated scaffolds. Additionally, rhBMP-2 released from the modified scaffolds enhanced alkaline phosphatase activity in W20–17 mouse bone marrow stromal cells, indicating the preservation of rhBMP-2 bioactivity indicative of osteogenesis.

**Conclusions:**

We conclude that this platform provides a sophisticated and efficient approach to deliver bioactive rhBMP-2 for bone tissue regeneration applications.

**Electronic supplementary material:**

The online version of this article (doi:10.1186/s40634-016-0057-1) contains supplementary material, which is available to authorized users.

## Background

Electrospun fiber meshes have gained increasing interest as tissue engineering scaffolds because of their nano- to microscale topography that resembles the native extracellular matrix (ECM) and their highly interconnected porosity, which facilitates nutrient and waste exchange (Cipitria et al. 2011; Lannutti et al. 2007). The process of electrospinning applies high voltage to a polymeric solution, generating electrostatic forces that drive the deposition of a non-woven fiber mesh consisting of solid polymeric fibers. The high specific surface area of these porous scaffolds renders them additionally useful for drug delivery applications (Sill and von Recum 2008). To date, a wide variety of natural and synthetic polymers such as collagen, fibrinogen, hyaluronic acid, poly(glycolic acid) and poly(ethylene-co-vinyl alcohol) have been electrospun to generate fibrous scaffolds for various tissue engineering applications (Hasan et al. 2014; Matthews et al. 2002; McManus et al. 2007; Pham et al. 2006b). As a biocompatible, biodegradable and low-cost synthetic polymer, poly(ε-caprolactone) (PCL) has emerged as one of the more widely investigated biomaterials for tissue engineering applications, including the regeneration of skin, nerve, and musculoskeletal tissues (Cipitria et al. 2011; Woodruff and Hutmacher 2010). Notably, electrospun PCL also has been actively explored as a platform for bone regeneration (Ekaputra et al. 2009; Liao et al. 2010; Mountziaris et al. 2013; Mountziaris et al. 2010; 2012; Thibault et al. 2010; Xie et al. 2013). However, because the material itself lacks inherent osteoinductive capacity, efforts have been undertaken to incorporate osteoinductive factors into the scaffold. Examples of such methods include coating the fiber surface with a bone-like ECM (Thibault et al. 2013) and enhancing local delivery of osteoinductive factors from within a biodegradable polymer (Martins et al. 2010). Among the known osteoinductive signaling factors, recombinant human bone morphogenetic proteins (rhBMPs) have been investigated most extensively as agents to encourage new bone formation. However, the short biological half-lives of these morphogens delivered as free compounds necessitate the use of supra-physiological doses to induce osteogenesis in the absence of a controlled-release delivery system. Such high levels can have serious adverse clinical repercussions, such as uncontrolled ectopic bone formation and inflammation (Haidar et al. 2009; Schmidmaier et al. 2008). To improve safety and reduce costs, rethinking the design of rhBMP delivery systems that increase osteoinductivity, while simultaneously achieving localized and controlled release of the delivered growth factor(s), is a critical undertaking (Haidar et al. 2009). Various immobilization mechanisms such as physical entrapment, adsorption, and complexation can be employed to sustain the long term delivery of non-covalently attached rhBMPs (Luginbuehl et al. 2004; Kim et al. 2014). Unfortunately, these methods often exacerbate the problem of potential growth factor inactivation (Luginbuehl et al. 2004). Heparin, a commercially available free glycosaminoglycan with structural-functional similarity to heparan sulfates found on proteoglycans, has gained wide use as a heparan sulfate mimetic to study glycosaminoglycan-growth factor interactions and to sequester growth factors in controlled delivery systems (Zhang 2010; Whitelock and Iozzo 2005). Heparin has been investigated as an adjunct to scaffolds to confer improved control of growth factor release kinetics (Zhang 2010; Jeon et al. 2007; Kim et al. 2011). Despite this, the actual native entities that bind, store, and activate this class of growth factors and morphogens are not heparin, but rather the polymeric heparan sulfate chains attached to proteoglycan core proteins present on cell surfaces and in the ECM (Zhang 2010). The biologically relevant interactions between morphogens and heparan sulfate are optimized by nature for both binding and release, and depend on the precise micropatterned structures of 2- and 6-O-sulfate moieties of heparan sulfate chains (Ashikari-Hada et al. 2004). To improve biologically relevant interactions both for binding and release by tissue heparanase, there is a shift away from using heparin as a global substitute in favor of more biologically appropriate forms of heparan sulfate (Whitelock and Iozzo 2005). Highly expressed in the human bone marrow and in other mesenchymal tissues, perlecan/HSPG2 is a large, secreted heparan sulfate proteoglycan with five distinct domains, each endowed with the unique ability to affect cellular processes such as cell binding, proliferation, differentiation, and angiogenesis (Knox and Whitelock 2006). Perlecan domain 1 (PlnD1) in particular, harbors three consensus glycosaminoglycan attachment sites that can contain up to three heparan sulfate chains for binding, storage, and release of heparin-binding growth factors and morphogens. Bound growth factors provide an “on demand” depot and are protected from denaturation or proteolytic degradation, while their biological activity can be augmented when released by natural enzymatic means such as heparanase activity (Decarlo et al. 2012; Knox and Whitelock 2006; Mongiat et al. 2001; Takada et al. 2003; McKeehan et al. 1999; Farach-Carson et al. 2014; Whitelock et al. 1996).

Given the ability of heparan sulfate-decorated PlnD1 to sequester and deliver rhBMPs and the need to utilize a more physiologic source of heparan sulfate, we hypothesized that conjugating PlnD1 to electrospun PCL scaffolds would increase both the growth factor loading capacity and the osteoinductivity of the scaffold. To test this, we developed a method to conjugate PlnD1 onto electrospun PCL fibers and tested the resulting modified PCL scaffold for increased binding and delivery of BMP-2 as well as bioactivity in inducing in vitro osteogenesis.

## Methods

### PlnD1 synthesis and purification

PInD1 was purified and characterized as described previously (Casper et al. 2007; Yang et al. 2006). PInD1 construct (amino acids 22–194) was designed for the mini-proteoglycan to be secreted into mammalian cell culture media for purification. Briefly, stably-transfected HEK 293 EBNA cells (Life Technologies, Carlsbad, CA) were cultured in high glucose Dulbecco’s Modified Eagle Medium (DMEM) (Life Technologies, Carlsbad, CA) supplemented with 10 % (v/v) heat-inactivated fetal bovine serum (FBS), 2 mM L-glutamine, 0.2 Units/mL penicillin, 0.2 μg/μL streptomycin (Life Technologies, Carlsbad, CA), 10 ng/mL puromycin, and 250 μg/mL geneticin (G418) (Life Technologies, Carlsbad, CA) and maintained at 37 °C in a 5 % CO_2_ incubator. Conditioned medium was prepared by culturing the cells in a HYPERflask® (Corning, Corning, NY) with DMEM supplemented with 2 % FBS. Conditioned medium that was collected was concentrated using a 10 kDa molecular weight cutoff spiral wound media concentrator. The concentrated conditioned medium was passed twice through a diethylaminoethyl (DEAE) column at 4 °C, washed extensively in a HEPES buffered solution (pH 8.0) containing 250 mM NaCl, 0.5 mM phenylmethylsulfonyl fluoride (PMSF), 0.5 mM benzamadine, and 0.2 % (w/v) sodium azide. PlnD1 was eluted from the column in a similar buffer containing 750 mM NaCl. The presence of PlnD1 was confirmed by assessing the fractions using a PlnD1-specific A76 and N-20 (Santa Cruz Biotechnology) antibody dot blot. Subsequently, PlnD1 was concentrated and exchanged into 25 mM HEPES, pH 7.4, 50 mM NaCl, and 1 % (v/v) glycerol solution using a 10 kDa molecular weight cutoff centrifugal filter (Millipore, Billerica, MA), 0.22 μm filtered, aliquoted, and stored at -80 °C. Purity was assessed through reducing SDS-PAGE (4–12 % acrylamide gradient gels (Life Technologies, Carlsbad, CA) in 3-(N-morpholino) propanesulfonic acid (MOPS) buffer), Coomassie staining and N-20 antibody western blots (Additional file 1: Figure S1). Approximately 20–30 mg at 2 mg/mL of pure PlnD1 was obtained from 4 L of conditioned medium.

### Fabrication of electrospun PCL scaffolds

As previously described, electrospun non-woven poly(ε-caprolactone) (Lactel, Birmingham, AL) mats (approximately 1 mm thickness) were fabricated with an average fiber diameter of approximately 10 μm (Pham et al. 2006a). Briefly, a syringe pump, power supply, and a grounded, square copper plate comprise the electrospinning setup. PCL (inherent viscosity range, 1.0–1.3) was dissolved in a 5:1 (v/v) chloroform/methanol solution to 18 % (w/w), and filled a 30-mL syringe fitted with a 16-gauge blunt needle. The needle and copper ring were connected via a split positive lead from the power supply. The electric field was stabilized by placing the copper ring between the needle and copper plate. A glass plate was placed in front of the copper plate to collect fibers during the electrospinning process. (Fong et al. 2013) Scanning electron microscopy (FEI Quanta 400 Environmental) was used to inspect the gold sputter-coated mats for consistent fiber morphology and diameter. Individual 3-mm scaffolds were then punched out of the mats using a dermal biopsy punch.

### Functionalization of PCL scaffolds with PlnD1

To conjugate PInD1 onto the surface of electrospun PCL fibers, the fabricated PCL scaffolds first were pre-wetted using an ethanol gradient starting with 100 % ethanol. ^125^I-labeled PInD1 was labeled by Perkin Elmer Life Sciences (Boston, MA) with >95 % purity and < 5 % free radiolabeled Iodine. Prewetted PCL fibers then were hydrolyzed with 0.5 M NaOH for 1 h and then protonated with 0.01 M HCl for 1 min to produce fiber surfaces bearing carboxylic groups. Scaffolds then were incubated in 0.1 M 2-(N-morpholino)ethanesulfonic acid (MES) (Sigma-Aldrich, St. Louis, MO) buffer for 1 h. Following this, the scaffolds were incubated in a buffer solution containing 7.8 mM sulfo-*N*-hydroxysulfosuccinimide (Sulfo-NHS) (Thermo Scientific, Rockville, IL), 39 mM 1-ethyl-3-(3-dimethylaminopropyl) carbodiimide hydrochloride (EDC) (Thermo Scientific, Rockville, IL), 0.5 M NaCl, and 0.1 M MES at pH 6.0 in the presence of ^125^I-labeled PInD1 for 3 h. Controls were placed into a similar buffer solution in the absence of Sulfo-NHS and EDC. Constructs were then washed 6 times with phosphate buffered saline (PBS), and the radioactivity of attached (covalent and non-specific) PlnD1 was measured using a gamma counter (Cobra II Autogamma, Packard, Meridian, CT). Constructs were next washed with 1 % (v/v) Tween-20 in PBS for 24 h with gentle shaking (70 rpm) to remove any non-covalently bound PlnD1, and radioactivity (DMPs) was measured again. Qualitative assessment of PlnD1 conjugation was performed by staining the constructs with Safranin O (1 mg/mL) overnight. Constructs were imaged using a digital camera (Nikon D2H) following a PBS rinse.

### rhBMP-2 binding and quantification of in vitro release kinetics

The release kinetics of rhBMP-2 from PInD1-conjugated PCL constructs was assessed by measuring the radioactivity of ^125^I-labeled rhBMP-2. Briefly, ^125^I-labeled rhBMP-2 (Perkin Elmer Life Sciences, Boston, MA) was incorporated with non-labeled rhBMP-2 (Peprotech, Rocky Hill, NJ) in 200 μL 3 % BSA in PBS (w/v) at a hot:cold ratio of 3:97. Constructs first were blocked with 3 % BSA in PBS (w/v) for 3 h at room temperature with gentle shaking (70 rpm) to minimize non-specific binding. Following which, each construct was incubated with the ^125^I-labeled and non-labeled rhBMP-2 solution (total of 4 μg rhBMP-2) overnight with gentle shaking (70 rpm) at 37 °C. After the incubation, constructs were washed thrice with 100 μL 3 % BSA in PBS (w/v) to remove any unattached rhBMP-2. The constructs each then were placed in a 5 ml culture tube (VWR, Radnor, PA), and a gamma counter was used to measure the initial amount of rhBMP-2 loaded within each construct. For 23 days, constructs were incubated with 1 mL of PBS with gentle shaking (70 rpm) at 37 °C. At days 1, 2, 5, 8, 11, 14, 17, 20, and 23 the supernatant of each construct was collected and replaced with fresh PBS. The amount of released growth factor was determined by the correlation of measured radioactivity (in dpms) to a standard curve using the gamma counter.

### Quantification of released rhBMP-2 bioactivity

To determine the in vitro biological activity of released rhBMP-2 from the PlnD1-conjugated PCL constructs, a previously reported method was employed with modifications (Kempen et al. 2008). This method is based on the W20–17 mouse bone marrow stromal cell line, which responds to rhBMP-2 in a dose-responsive manner by increasing alkaline phosphatase activity (Thies et al. 1992). Over a period of 23 days, medium that was incubated with the rhBMP-2-releasing PlnD1-conjugated PCL constructs (or unmodified PCL scaffolds) for 1 day (for the first two time-points) or 3 days (for the rest of the time-points) was transferred to fresh W20–17 cell cultures at designated time-points to determine the levels of alkaline phosphatase activity.

Cells were cultured in Dulbecco’s Modified Eagle’s Medium (DMEM) containing 10 % (v/v) fetal bovine serum and 1 % (from stock) antibiotics/antimycotics. Prior to the start of the in vitro experiment, W20–17 cells were expanded and cryopreserved in multiple aliquots. To establish new cultures for each time-point, an aliquot (passage 3) was thawed, expanded for 3 days, and re-plated in 24-well plates at a density of 20,000 cells/cm^2^. Medium was replaced the next day with medium that had been exposed to the rhBMP-2-releasing PlnD1-conjugated PCL constructs (or unmodified PCL scaffolds) from the previous time-point, and cells were incubated with this medium for 3 days. W20–17 cells were treated with medium containing 0, 10, 50, 100 or 500 ng/mL of rhBMP-2 to verify the dose-responsive effect of rhBMP-2 on alkaline phosphatase activity (positive controls). The collected samples underwent three cycles of freezing and thawing, and then were ultrasonicated to lyse the cells. The cell lysates were subsequently assayed for cellularity and alkaline phosphatase activity. Cellularity was determined by using the Quant-iT™ PicoGreen® dsDNA assay kit (Invitrogen™) as per the manufacturer’s instructions. Briefly, cell lysate, assay buffer, and dye solution were mixed and allowed to incubate for 10 min at room temperature. Excitation and emission wavelengths of 485 and 528 nm, respectively, were used to measure the fluorescence (FLx800 fluorescence microplate reader; BioTek Instruments). A lambda DNA standard curve was used to determine DNA concentrations. Alkaline phosphatase activity was measured using alkaline buffer solution and phosphatase substrate tablets (Sigma). Briefly, cell lysate and the reagents were mixed and incubated at 37 °C for 1 h. NaOH was used to stop the reaction, and absorbance at 405 nm was measured (PowerWave x340 Microplate Reader; BioTek Instruments). A p-nitrophenol standard curve was used to determine alkaline phosphatase activity, which then was normalized to DNA content for each sample.

### Statistical analysis

Data are presented as mean ± standard deviation for *n* = 3 throughout the study. One-way analysis of variance (ANOVA) followed by Tukey’s multiple-comparison test was used in the analysis of data. *p* < 0.05 was considered to indicate a significant difference.

## Results

### Covalent modification of PCL fiber surfaces and quantification of bound PlnD1

**Fig. 1:**
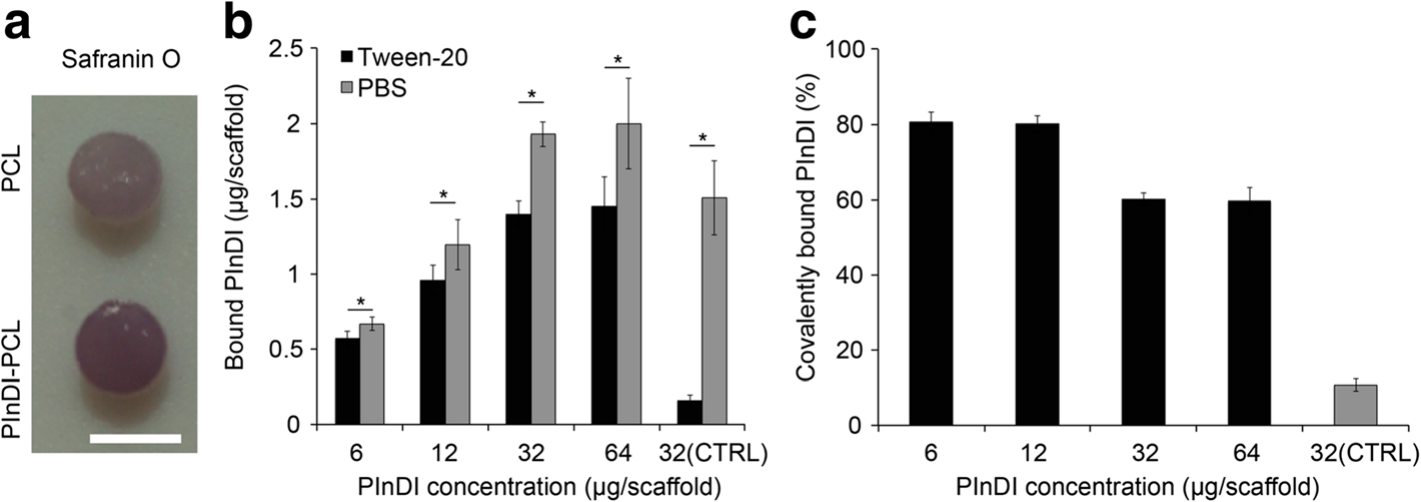
Covalently conjugating PlnD1 to PCL scaffolds. Panel **a** shows qualitative confirmation of PlnD1 conjugation to PCL via Safranin O staining. Scaffold on the bottom was processed with Sulfo-NHS/EDC, which facilitated the reaction of free carboxylic groups on PCL with amines on PlnD1. Scaffold on the top was unmodified. To quantitatively determine the maximum amount of PlnD1 that could be conjugated to the PCL scaffold, scaffolds were incubated with 6.4, 19.2, 32, or 64 μg of PlnD1 per scaffold. PBS was used in place of NHS/EDC in the conjugation reaction for the control (CTRL) group. Panel **b** shows the amount of PlnD1 detected following a PBS wash, followed by another wash with 1 % Tween-20. The latter was employed to remove any non-specific binding of PlnD1 on the scaffold. **c** Covalently bound PInDI in PCL was defined as amount of PlnD1 left on the scaffold after the Tween-20 wash (covalently conjugated PlnD1) divided by the amount of PlnD1 on the scaffold after the PBS wash (includes both covalently conjugated and non-specific bound PlnD1) x 100 %. (*n* = 3) Error bars correspond to standard deviation. Scale bar = 3 mm in (**a**). (*) indicates a statistical difference between groups (*p* < 0.05)

Following a previously established method, reactive groups were generated on electrospun PCL fiber surfaces via base-catalyzed hydrolysis of the ester bond in the PCL backbone followed by conversion of the resulting carboxylates to carboxylic acid groups with hydrochloric acid (Hartman et al. 2010). Using sulfo-NHS/EDC-mediated chemistry, PlnD1 then was coupled to the carboxylated PCL via free amines within the peptide. Given the presence of glycosaminoglycan chains on PlnD1, conjugation of the peptide onto PCL was confirmed by staining the constructs with Safranin O in deionized water (Kiviranta et al. 1985). As shown in Fig. 1a, the darker stained construct corresponded to scaffolds that were treated with EDC/NHS, indicating the presence of more glycosaminoglycans and hence, covalently conjugated PlnD1. Notably, Tween-20 was used to remove any non-covalently bound PlnD1 prior to the staining.

To quantify this conjugation, scaffolds were incubated with varying amounts of ^125^I-labeled PInD1, and the radioactivity of the resulting PlnD1-bound constructs was measured. In the presence of increasing amounts of ^125^I-labeled PInD1, a corresponding increase in the amount of ^125^I-labeled PInD1 was measured for each construct (Fig. 1b). Notably, no further increase in the amount of PlnD1 was observed beyond 32 μg of the peptide in the incubation buffer solution. As non-specific binding of PlnD1 to the modified PCL surfaces also could occur, the constructs were treated further with Tween-20 to remove any non-covalently conjugated PlnD1 before radioactivity was measured again. A similar trend of increasing construct-associated ^125^I-labeled PInD1 with increasing amounts of ^125^I-labeled PInD1 in the incubation buffer solution was observed, although at lower levels, indicating that covalent binding accounts for the attachment of PlnD1 to the modified PCL surfaces. This difference in the amount of ^125^I-labeled PInD1 detected before and after treatment with Tween-20 was expressed as the percentage of PlnD1 retained via covalent coupling to the PCL surface. Again, no further increase in the amount of covalently bound ^125^I-labeled PInD1 was observed beyond 32 μg, where the percentage of PlnD1 retained was approximately 60 % (Fig. 1c), suggesting that maximum loading of PlnD1 onto the modified PCL scaffold could be achieved in the presence of 32 μg of PlnD1 in the incubation buffer solution.

### Quantification of rhBMP-2 loading

**Fig. 2:**
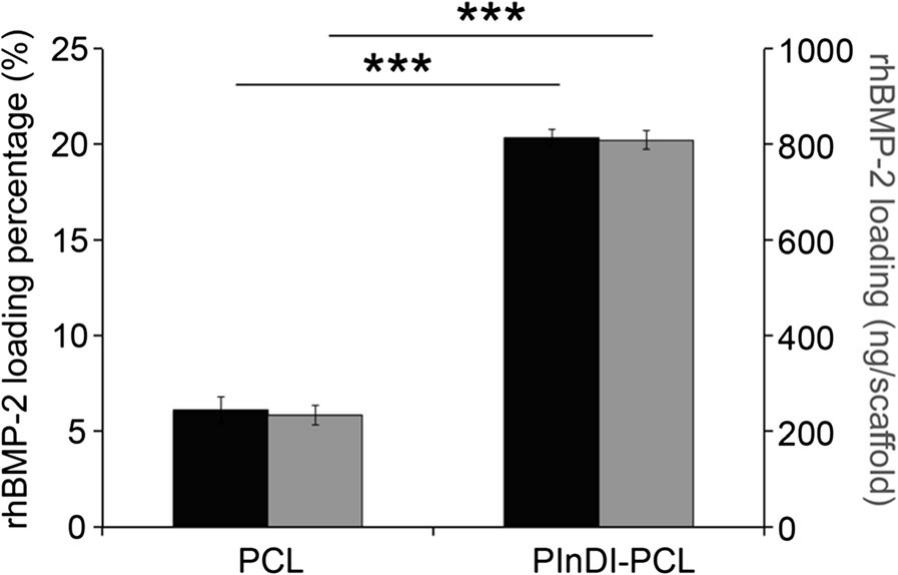
PInDI-PCL scaffolds increased rhBMP-2 loading. rhBMP-2 loading efficiency and amount of PlnD1-modified and unmodified PCL scaffolds following an overnight incubation of the scaffolds with 4 μg of rhBMP-2. Error bars correspond to standard deviation (*n* = 3). (***) indicates a statistical difference between groups (*p* < 0.001)

After confirming that PlnD1 was covalently coupled to the electrospun PCL fibers, we next sought to determine if the presence of PlnD1 on the PCL fiber surface resulted in a higher rhBMP-2 loading capacity as compared to the unmodified PCL scaffolds. PlnD1-conjugated constructs and unmodified PCL scaffolds were incubated with the same amount of ^125^I-labeled rhBMP-2 (4 μg), and the radioactivity of the resulting rhBMP-2-loaded constructs was measured. Loading capacity was defined as the ratio of the detected rhBMP-2 in the PlnD1-conjugated constructs or unmodified PCL scaffolds over the total rhBMP-2 initially present in the incubation solution. As shown in Fig. 2, PlnD1-conjugated PCL constructs exhibited a four-fold higher rhBMP-2 loading capacity (20 %) as compared to the unmodified PCL scaffolds (5 %), underscoring the ability of the heparan sulfate chains on the conjugated PlnD1 to sequester the growth factor and increase rhBMP-2 loading efficiency relative to unmodified scaffolds. PlnD1-conjugated PCL constructs initially bound 809 ± 19 ng of rhBMP-2 in contrast with PCL only scaffolds that bound 233 ± 21 ng of rhBMP-2.

### Quantification of rhBMP-2 release kinetics

**Fig. 3:**
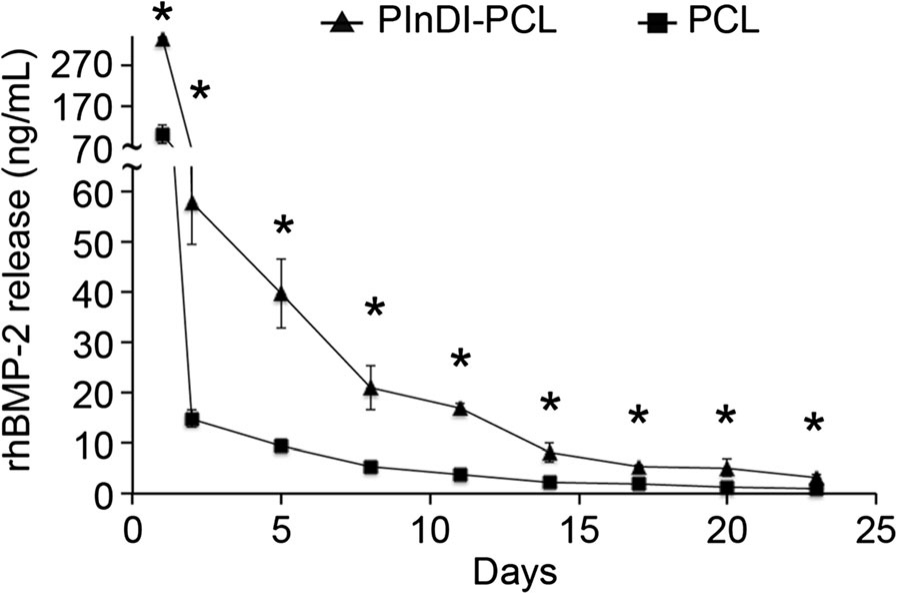
PInDI modified scaffolds controlled rhBMP-2 release. The absolute amount of rhBMP-2 released from PlnD1-conjugated or unmodified PCL scaffolds over 23 days. (*n* = 3) Error bars correspond to standard deviation. (*) indicates a statistical difference between groups (*p* < 0.05)

The absolute amount (Fig. 3) and cumulative release (Additional file 1: Figure S3) profile of rhBMP-2 (as measured by radioactive ^125^I-labeled rhBMP-2) over 23 days of either the PlnD1-conjugated PCL constructs or unmodified PCL scaffolds shown that PlnD1-conjugated PCL constructs provided sustained release of rhBMP-2 in PBS. The amount of rhBMP-2 released by the PlnD1-conjugated PCL constructs was statistically higher than that of the unmodified PCL scaffolds at all time-points.

### Bioactivity of released rhBMP-2

**Fig. 4:**
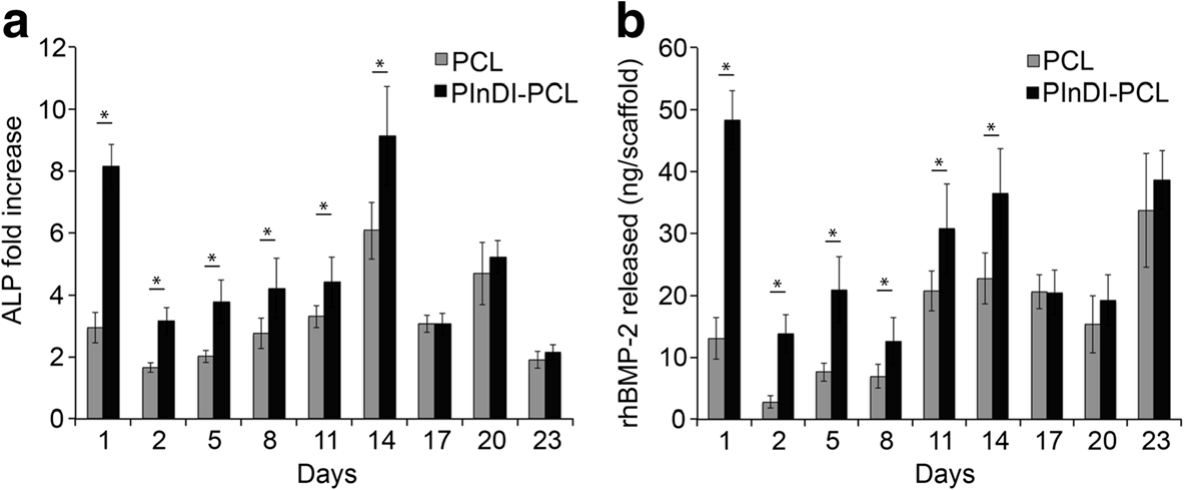
PInDI-PCL scaffold preserved rhBMP-2 bioactivity. **a** ALP activity of W20–17 cultures exposed to rhBMP-2 released from either PlnD1-conjugated or unmodified PCL scaffolds. Fresh W20–17 cultures were used for each time-point. ALP activity was normalized to DNA content and expressed as a fold-change over basal ALP activity. **b** Amount of bioactive rhBMP-2 released from PlnD1-modified or unmodified PCL scaffolds as measured by comparing against a rhBMP-2 dose response curve generated by adding rhBMP-2 directly to W20–17 cultures (positive control). (*n* = 5) Error bars correspond to standard deviation. (*) indicates a statistical difference between groups (*p* < 0.05)

The bioactivity of rhBMP-2 released from the PlnD1-conjugated PCL constructs was determined by measuring the ability of the released growth factor to induce ALP activity in the W20–17 mouse bone marrow stromal cells. Fresh cultures were used for each time-point for the evaluation. Figure 4a depicts the fold change in DNA-normalized ALP over basal ALP levels in the W20–17 cells. rhBMP-2 released from the PlnD1-conjugated PCL constructs induced a significantly higher ALP activity over basal levels as compared to the unmodified PCL scaffolds up to 14 days. However, there is no difference in W20–17 proliferation between groups (Additional file 1: Figure S3). Using a standard curve generated by exposing the W20–17 cells to known amounts of rhBMP-2 in culture medium, the amount of bioactive rhBMP-2 released at each time-point was obtained, as shown in Fig. 4b. According to this method of detection, PlnD1-conjugated PCL constructs released significantly more bioactive rhBMP-2 than unmodified PCL scaffolds up to day 14. There is a modest difference at day 20 and day 23.

## Discussion

Although rhBMP-2 and rhBMP-7 are available for use in the clinic for orthopedic regenerative procedures, their use is generally currently limited by high costs and the need for supra-physiologic levels, as well as inadequate control over bone healing and a risk of inflammation (Luginbuehl et al. 2004; DeCarlo and Whitelock 2006; Lee et al. 2012). To address these problems, efforts have been directed towards developing rhBMP delivery systems capable of localizing and modulating the release of the morphogenetic stimulus for safe and consistent clinical success. While growth factor delivery strategies based on physical adsorption, ionic complexation, or covalent immobilization have been investigated, these approaches are generally associated with the risk of undesired reduction in bioactivity and bolus induced attraction of inflammatory cells. An alternative strategy is to harness the innate function of heparan sulfate proteoglycans in the native ECM to sequester and modulate the availability and activity of morphogens or growth factors such as rhBMP-2 (DeCarlo and Whitelock 2006). While this approach has been investigated in the form of heparin incorporation to scaffolds, such as those based on poly(L-lactic-co-glycolic acid) (Jeon et al. 2007), chitosan (Engstrand et al. 2008), fibrin (Yang et al. 2010) and PCL (Kim et al. 2014) to deliver rhBMP-2, the use of heparin to deliver growth factors has limited physiological relevance. Moreover, the effect of heparin on rhBMP-2 biological activity is mixed–while it has been reported to enhance the biological activity of rhBMP-2, it also has been reported to inhibit rhBMP-2 binding to its receptor and reduce rhBMP-2 osteogenic signaling, underscoring the need to review the suitability of using heparin as a rhBMP-2 carrier (Kanzaki et al. 2008; Jiao et al. 2007; Takada et al. 2003). Moreover, heparin has limited susceptibility to heparanase digestion (Meikle et al. 2005).

Yet to be fully appreciated, the entity that stores, stabilizes, and presents growth factors in more active configurations to their receptors is not heparin, but rather the heparan sulfate chains associated with proteoglycans on cell surfaces and the ECM (Decarlo et al. 2012; Casu et al. 2010). One such proteoglycan is perlecan/HSPG2, a highly conserved ECM component in bone vasculature and bone marrow stroma, which is also associated with bone healing, as the perlecan gene was reported to be one of the earliest genes expressed in new fracture callous formation in fractured bone (Wang et al. 2006; Farach-Carson et al. 2014). Leveraging the prevalence of perlecan in bone healing, the physiological relevance of using heparan sulfate-decorated PlnD1 to sequester and release rhBMP-2 and the previously demonstrated utility of electrospun PCL scaffolds for bone regeneration, we have developed a novel rhBMP-2 delivery system for bone regeneration applications. An injectable form of this combined with hyaluronan was recently shown by our group to potentiate the cartilage repair effect of rhBMP-2 in an experimental model of osteoarthritis (Srinivasan et al. 2012).

The goal of this study was to determine if functionalization of electrospun PCL fibers with PlnD1 enhances rhBMP-2 binding and if subsequently released rhBMP-2 presents bioactivity. To achieve this, we employed a previously reported method from our laboratory (Hartman et al. 2010) to conjugate proteins onto PCL surfaces (i.e., via the introduction of carboxylate groups to surface-hydrolyzed PCL and the use of EDC/NHS chemistry) and demonstrated that the presence of covalently attached PlnD1 indeed increases the rhBMP-2 loading capacity of electrospun PCL scaffolds and bioactive release as compared to controls with physically adsorbed rhBMP-2. We first fabricated PlnD1-conjugated electrospun PCL constructs and compared the rhBMP-2 loading efficiency to that of unmodified PCL. In the presence of conjugated PlnD1, the rhBMP-2 loading efficiency was approximately four-fold higher, indicating the ability of PlnD1 to significantly enhance the loading capacity of electrospun PCL scaffolds. This increase likely is reflective of the increase in the number of rhBMP-2 binding sites due to multivalency in the presence of the three heparan sulfate chains in PlnD1 (Jha et al. 2009). That covalently conjugated PlnD1 increases the rhBMP-2 binding capacity of PCL scaffolds and mirrors the findings of a previous study in which rhBMP-2 loading onto hyaluronan hydrogel microparticles was augmented in the presence of PlnD1 (Jha et al. 2009). Furthermore, with the use of heparitinase, it was demonstrated that rhBMP-2 binding was heparan sulfate-dependent (Jha et al. 2009). Comparing the increase in rhBMP-2 binding capacity due to PlnD1 in this study to one of the two major methods of attaching growth factors or morphogens onto the surface of scaffolds–chemical conjugation directly onto the material surface (the other being physical adsorption)–we found that this increase in rhBMP-2 binding capacity due to PlnD1 is comparable to that reported by Zhang et al., where rhBMP-2 was chemically conjugated directly onto PCL scaffolds (approximately 4-fold higher than physically adsorbed rhBMP-2).(Zhang et al. 2010) Notably, beyond the indication that the use of PlnD1 to augment rhBMP-2 loading is as effective as chemically conjugating the growth factor directly onto the material surface, the heparan sulfate chains may provide the additional beneficial effect of potentiating the bioactivity of the delivered rhBMP-2, not achievable with just delivery of the growth factor alone.

While the presence of PlnD1 resulted in a higher rhBMP-2 loading capacity as compared to plain unmodified PCL, the release pattern of the growth factor was similar between the two groups. The release kinetics were characterized by a rapid initial release followed by a slow, sustained release over 23 days. By covalent coupling of PlnD1 to hyaluronan microparticles, it was previously demonstrated that the presence of PlnD1 diminished the initial burst release of rhBMP-2 from non-functionalized microparticles, resulting in a more linear and sustained rhBMP-2 release.(Jha et al. 2009) In another study where electrospun PCL fibers were modified with heparin-dopamine for the delivery of rhBMP-2, the authors reported that the presence of heparin resulted in the absence of a high initial burst release of the growth factor. However, the release kinetics of rhBMP-2 from plain unmodified PCL was not presented for comparison against the heparin-modified constructs (Kim et al. 2014). These studies and others suggest that the presence of heparan sulfate-decorated PlnD1 or heparin is associated with a dampened initial growth factor release. However, in our study, while enhanced rhBMP-2 loading was observed, a reduction in initial release kinetics was not, in the presence of PlnD1. This is potentially due to the presence of physically adsorbed PlnD1, which can also bind rhBMP-2 in addition to the covalently conjugated PlnD1. Upon incubation, the immediate desorption of physically adsorbed, rhBMP-2-binding PlnD1 from the PCL surface could have contributed to the initial burst release. Accordingly, because the ability of PlnD1 to sequester rhBMP-2 may have been masked by the desorption of physically adsorbed PlnD1, the release kinetics of rhBMP-2 from PlnD1-conjugated and plain unmodified PCL scaffolds are not different. It is noteworthy though that only 59.7 ± 1.2 % rhBMP-2 in the loading solution was released at the end of 23 days in this study. As discussed above, the amount of rhBMP-2 released from PlnD1-conjugated microparticles was close to 70 % at the end of 15 days while in another study where rhBMP-2 was coated onto polystyrene/PCL fibers, the amount of rhBMP-2 released after 2 weeks was also approximately 70 %. Although these systems differ, these comparisons indicate that PlnD1-conjugated PCL constructs are capable of long-term retention of rhBMP-2; the remaining rhBMP-2 measured at end time-point could potentially be released with extended incubation, or by the activity of heparanases (Jha et al. 2009).

Using the W20–17 mouse bone marrow stromal cell line, the bioactivity of released BMP-2 from the PlnD1-conjugated PCL constructs and unmodified PCL scaffolds was assessed by determining the ability of the released growth factor to induce ALP activity over basal levels in the W20–17 cells and comparing the detected ALP levels to a standard curve generated by exposing the cells to known amounts of rhBMP-2. Even though the amount of rhBMP-2 released from the PlnD1-conjugated constructs was significantly greater than the unmodified PCL scaffolds at all time-points (up to day 23), ALP activity induced in the W20–17 cultures was only significantly higher up to day 14. The biphasic profile often is observed in many growth factor release systems in which simple diffusion governs the growth factor release (Jha et al. 2009). This is likely because this assay may have limited sensitivity at the later time-points where the differences in BMP-2 released between the groups are small. Additionally, the release kinetics in serum might be slightly different than PBS since high protein environment might interfere with the protein bindings. Given the ability of the BMP-2 released to induce an increase in ALP activity over basal levels in the W20–17 cells in both systems, this indicates that the released protein is stable and that structural integrity is maintained (Kempen et al. 2008). By conjugating heparin-dopamine onto PCL fibers to deliver rhBMP-2, Kim et al. reported that the rhBMP-2 binding heparin-conjugated PCL fibers could significantly induce greater osteogenic differentiation in periodontal ligament cells relative to PCL fibers alone, corroborating the findings of our study. Notably, the PCL-only control in this aforementioned study was not physically adsorbed with rhBMP-2, and cells were seeded directly onto the scaffolds (Kim et al. 2014). Taken together, these results indicate that rhBMP-2 released from PlnD1-functionalized PCL fibers maintains structural integrity and bioactivity necessary to confer osteoinductive properties to PCL fiber scaffolds.

## Conclusions

In this study, we report a novel method to efficiently covalently conjugate heparan sulfate-decorated PlnD1 to the surface of electrospun PCL fibers for rhBMP-2 binding and controlled release. Covalently conjugated heparan sulfate-decorated PlnD1 significantly increased the loading capacity and retention of rhBMP-2 in electrospun PCL scaffolds and subsequently maintained the in vitro osteogenic activity of the released growth factor. The increased loading capacity of the PCL scaffold in the presence of PlnD1 underscores the potential for use of rhBMP-2 in tissue engineering applications. More importantly, we demonstrate that in place of heparin, physiologically relevant heparan sulfate-decorated PlnD1 is a useful adjunct to PCL scaffolds for rhBMP-2 delivery and potential enhancement of bioactivity for bone tissue regeneration without adverse effects of high local concentrations of rhBMP-2.

## Additional file

Additional file 1: Figure S1. Perlecan domain I (Dm1) purification and glycosaminoglycan characterization. Perlecan Dm1 purified from HEK293 cells was incubated alone (lanes 2, 9) or with heparitinases 1, 2, 3 and chondroitinase ABC either together (lanes 3, 10) or separately (lanes 5–8). Lane 1 is the molecular weight marker and Lane 4 is all enzymes without Dm1. On the left is a Coomassie stain (lanes 1–8) and on the right is a western blot (lanes 9–10) using a domain I specific antibody (N-20). The arrow head indicates the glycosylated form of Dm I and the arrow indicates the protein core. All enzymes were incubated at 0.1 Units per 10 μg of Dm 1 in a 20 μL of reaction at 37 °C for 4 h. The buffer was 20 mM Tris-HCl, 10 mM NaCl, and 3 mM calcium acetate at pH 8.0. **Figure S2.** PInDI modified scaffolds controlled rhBMP-2 cumulative release. The absolute amount of rhBMP-2 released from PlnD1-conjugated or unmodified PCL scaffolds over 23 days. (*n* = 3) Error bars correspond to standard deviation. **Figure S3.** DNA concentration of W20–17 cells after exposing to rhBMP-2 released from either PlnD1-conjugated or unmodified PCL scaffolds. Fresh W20–17 cultures were used for each time-point (*n* = 5). (DOCX 227 kb)

## Abbreviations

ALP: Alkaline phosphatase
DEAE: Diethylaminoethyl
DMEM: Dulbecco’s modified eagle’s medium
DMPs: Radioactivity
EDC: Carbodiimide hydrochloride
FBS: Fetal bovine serum
MES: 2-(N-morpholino)ethanesulfonic acid
PBS: Phosphate buffered saline
PCL: Poly(ε-caprolactone)
PlnD1: Perlecan domain 1
PMSF: Phenylmethylsulfonyl fluoride
rhBMPs: Recombinant human bone morphogenetic proteins
Sulfo-NHS: Sulfo-*N*-hydroxysulfosuccinimide

## References

[CR1] Ashikari-Hada S, Habuchi H, Kariya Y, Itoh N, Reddi AH, Kimata K (2004). Characterization of growth factor-binding structures in heparin/heparan sulfate using an octasaccharide library. J Biol Chem.

[CR2] Casper CL, Yang W, Farach-Carson MC, Rabolt JF (2007). Coating electrospun collagen and gelatin fibers with perlecan domain I for increased growth factor binding. Biomacromolecules.

[CR3] Casu B, Naggi A, Torri G (2010). Heparin-derived heparan sulfate mimics to modulate heparan sulfate-protein interaction in inflammation and cancer. Matrix Biol.

[CR4] Cipitria A, Skelton A, Dargaville TR, Dalton PD, Hutmacher DW (2011). Design, fabrication and characterization of PCL electrospun scaffolds-a review. J Mater Chem.

[CR5] DeCarlo AA, Whitelock JM (2006). The role of heparan sulfate and perlecan in bone-regenerative procedures. J Dental Res.

[CR6] Decarlo AA, Belousova M, Ellis AL, Petersen D, Grenett H, Hardigan P, O’Grady R, Lord M, Whitelock JM (2012). Perlecan domain 1 recombinant proteoglycan augments BMP-2 activity and osteogenesis. BMC Biotechnol.

[CR7] Ekaputra AK, Zhou Y, Cool SM, Hutmacher DW (2009). Composite electrospun scaffolds for engineering tubular bone grafts. Tissue Eng Part A.

[CR8] Engstrand T, Veltheim R, Arnander C, Docherty-Skogh AC, Westermark A, Ohlsson C, Adolfsson L, Larm O (2008). A novel biodegradable delivery system for bone morphogenetic protein-2. Plast Reconstr Surg.

[CR9] Farach-Carson MC, Warren CR, Harrington DA, Carson DD (2014). Border patrol: Insights into the unique role of perlecan/heparan sulfate proteoglycan 2 at cell and tissue borders. Matrix Biol.

[CR10] Fong EL, Lamhamedi-Cherradi SE, Burdett E, Ramamoorthy V, Lazar AJ, Kasper FK, Farach-Carson MC, Vishwamitra D, Demicco EG, Menegaz BA, Amin HM, Mikos AG, Ludwig JA (2013). Modeling Ewing sarcoma tumors in vitro with 3D scaffolds. Proc Natl Acad Sci U S A.

[CR11] Haidar ZS, Hamdy RC, Tabrizian M (2009). Delivery of recombinant bone morphogenetic proteins for bone regeneration and repair. Part A: Current challenges in BMP delivery. Biotechnol Lett.

[CR12] Hartman O, Zhang C, Adams EL, Farach-Carson MC, Petrelli NJ, Chase BD, Rabolt JF (2010). Biofunctionalization of electrospun PCL-based scaffolds with perlecan domain IV peptide to create a 3-D pharmacokinetic cancer model. Biomaterials.

[CR13] Hasan A, Memic A, Annabi N, Hossain M, Paul A, Dokmeci MR, Dehghani F, Khademhosseini A (2014). Electrospun scaffolds for tissue engineering of vascular grafts. Acta Biomater.

[CR14] Jeon O, Song SJ, Kang SW, Putnam AJ, Kim BS (2007). Enhancement of ectopic bone formation by bone morphogenetic protein-2 released from a heparin-conjugated poly(L-lactic-co-glycolic acid) scaffold. Biomaterials.

[CR15] Jha AK, Yang W, Kirn-Safran CB, Farach-Carson MC, Jia X (2009). Perlecan domain I-conjugated, hyaluronic acid-based hydrogel particles for enhanced chondrogenic differentiation via BMP-2 release. Biomaterials.

[CR16] Jiao X, Billings PC, O’Connell MP, Kaplan FS, Shore EM, Glaser DL (2007). Heparan sulfate proteoglycans (HSPGs) modulate BMP2 osteogenic bioactivity in C2C12 cells. J Biol Chem.

[CR17] Kanzaki S, Takahashi T, Kanno T, Ariyoshi W, Shinmyouzu K, Tujisawa T, Nishihara T (2008). Heparin inhibits BMP-2 osteogenic bioactivity by binding to both BMP-2 and BMP receptor. J Cell Physio.

[CR18] Kempen DH, Lu L, Hefferan TE, Creemers LB, Maran A, Classic KL, Dhert WJ, Yaszemski MJ (2008). Retention of in vitro and in vivo BMP-2 bioactivities in sustained delivery vehicles for bone tissue engineering. Biomaterials.

[CR19] Kim SE, Song SH, Yun YP, Choi BJ, Kwon IK, Bae MS, Moon HJ, Kwon YD (2011). The effect of immobilization of heparin and bone morphogenic protein-2 (BMP-2) to titanium surfaces on inflammation and osteoblast function. Biomaterials.

[CR20] Kim SE, Yun Y-P, Han Y-K, Lee D-W, Ohe J-Y, Lee B-S, Song H-R, Park K, Choi B-J (2014). Osteogenesis induction of periodontal ligament cells onto bone morphogenic protein-2 immobilized PCL fibers. Carbohydr Polym.

[CR21] Kiviranta I, Jurvelin J, Tammi M, Saamanen AM, Helminen HJ (1985). Microspectrophotometric quantitation of glycosaminoglycans in articular cartilage sections stained with Safranin O. Histochemistry.

[CR22] Knox SM, Whitelock JM (2006). Perlecan: how does one molecule do so many things?. Cell Mol Life Sci.

[CR23] Lannutti J, Reneker D, Ma T, Tomasko D, Farson DF (2007). Electrospinning for tissue engineering scaffolds. Mat Sci Eng C-Bio S.

[CR24] Lee KB, Taghavi CE, Murray SS, Song KJ, Keorochana G, Wang JC (2012). BMP induced inflammation: a comparison of rhBMP-7 and rhBMP-2. J Orthop Res.

[CR25] Liao J, Guo X, Nelson D, Kasper FK, Mikos AG (2010). Modulation of osteogenic properties of biodegradable polymer/extracellular matrix scaffolds generated with a flow perfusion bioreactor. Acta Biomater.

[CR26] Luginbuehl V, Meinel L, Merkle HP, Gander B (2004). Localized delivery of growth factors for bone repair. Eur J Pharm Biopharm.

[CR27] Martins A, Duarte AR, Faria S, Marques AP, Reis RL, Neves NM (2010). Osteogenic induction of hBMSCs by electrospun scaffolds with dexamethasone release functionality. Biomaterials.

[CR28] Matthews JA, Wnek GE, Simpson DG, Bowlin GL (2002). Electrospinning of collagen nanofibers. Biomacromolecules.

[CR29] McKeehan WL, Wu X, Kan M (1999). Requirement for anticoagulant heparan sulfate in the fibroblast growth factor receptor complex. J Biol Chem.

[CR30] McManus MC, Boland ED, Simpson DG, Barnes CP, Bowlin GL (2007). Electrospun fibrinogen: Feasibility as a tissue engineering scaffold in a rat cell culture model. J Biomed Mater Res A.

[CR31] Meikle PJ, Fuller M, Hopwood JJ (2005) Chemistry and Biology of Heparin and Heparan Sulfate. Elsevier B.V. doi:10.1016/B978-008044859-6/50011-3

[CR32] Mongiat M, Otto J, Oldershaw R, Ferrer F, Sato JD, Iozzo RV (2001). Fibroblast growth factor-binding protein is a novel partner for perlecan protein core. J Biol Chem.

[CR33] Mountziaris PM, Tzouanas SN, Mikos AG (2010). Dose effect of tumor necrosis factor-alpha on in vitro osteogenic differentiation of mesenchymal stem cells on biodegradable polymeric microfiber scaffolds. Biomaterials.

[CR34] Mountziaris PM, Tzouanas SN, Mikos AG (2012). Student Award for Outstanding Research Winner in the Ph.D. Category for the 9th World Biomaterials Congress, Chengdu, China, June 1–5, 2012: The interplay of bone-like extracellular matrix and TNF-alpha signaling on in vitro osteogenic differentiation of mesenchymal stem cells. J Biomed Mater Res A.

[CR35] Mountziaris PM, Dennis Lehman E, Mountziaris I, Sing DC, Kasper FK, Mikos AG (2013). Effect of temporally patterned TNF-alpha delivery on in vitro osteogenic differentiation of mesenchymal stem cells cultured on biodegradable polymer scaffolds. J Biomater Sci Polym Ed.

[CR36] Pham QP, Sharma U, Mikos AG (2006a) Electrospinning of polymeric nanofibers for tissue engineering applications: a review. Tissue Eng. 12(5):1197-1211. doi:10.1089/ten.2006.12.1197 10.1089/ten.2006.12.1197 16771634

[CR37] Pham QP, Sharma U, Mikos AG (2006b) Electrospinning of polymeric nanofibers for tissue engineering applications: A review. Tissue Eng 12(5):1197-1211. doi: 10.1089/Ten.2006.12.1197 10.1089/ten.2006.12.1197 16771634

[CR38] Schmidmaier G, Schwabe P, Strobel C, Wildemann B (2008). Carrier systems and application of growth factors in orthopaedics. Injury.

[CR39] Sill TJ, von Recum HA (2008). Electrospinning: applications in drug delivery and tissue engineering. Biomaterials.

[CR40] Srinivasan PP, McCoy SY, Jha AK, Yang W, Jia X, Farach-Carson MC, Kirn-Safran CB (2012). Injectable perlecan domain 1-hyaluronan microgels potentiate the cartilage repair effect of BMP2 in a murine model of early osteoarthritis. Biomed Mater.

[CR41] Takada T, Katagiri T, Ifuku M, Morimura N, Kobayashi M, Hasegawa K, Ogamo A, Kamijo R (2003). Sulfated polysaccharides enhance the biological activities of bone morphogenetic proteins. J Biol Chem.

[CR42] Thibault RA, Scott Baggett L, Mikos AG, Kasper FK (2010). Osteogenic differentiation of mesenchymal stem cells on pregenerated extracellular matrix scaffolds in the absence of osteogenic cell culture supplements. Tissue Eng Part A.

[CR43] Thibault RA, Mikos AG, Kasper FK (2013). Winner of the 2013 Young Investigator Award for the Society for Biomaterials annual meeting and exposition, April 10–13, 2013, Boston, Massachusetts. Osteogenic differentiation of mesenchymal stem cells on demineralized and devitalized biodegradable polymer and extracellular matrix hybrid constructs. J Biomed Mater Res A.

[CR44] Thies RS, Bauduy M, Ashton BA, Kurtzberg L, Wozney JM, Rosen V (1992). Recombinant human bone morphogenetic protein-2 induces osteoblastic differentiation in W-20–17 stromal cells. Endocrinology.

[CR45] Wang K, Vishwanath P, Eichler GS, Al-Sebaei MO, Edgar CM, Einhorn TA, Smith TF, Gerstenfeld LC (2006). Analysis of fracture healing by large-scale transcriptional profile identified temporal relationships between metalloproteinase and ADAMTS mRNA expression. Matrix Biol.

[CR46] Whitelock JM, Iozzo RV (2005). Heparan sulfate: a complex polymer charged with biological activity. Chem Rev.

[CR47] Whitelock JM, Murdoch AD, Iozzo RV, Underwood PA (1996). The degradation of human endothelial cell-derived perlecan and release of bound basic fibroblast growth factor by stromelysin, collagenase, plasmin, and heparanases. J Biol Chem.

[CR48] Woodruff MA, Hutmacher DW (2010). The return of a forgotten polymer-Polycaprolactone in the 21st century. Prog Polym Sci.

[CR49] Xie J, Zhong S, Ma B, Shuler FD, Lim CT (2013). Controlled biomineralization of electrospun poly(epsilon-caprolactone) fibers to enhance their mechanical properties. Acta Biomater.

[CR50] Yang W, Gomes RR, Brown AJ, Burdett AR, Alicknavitch M, Farach-Carson MC, Carson DD (2006). Chondrogenic differentiation on perlecan domain I, collagen II, and bone morphogenetic protein-2-based matrices. Tissue Eng.

[CR51] Yang HS, La WG, Bhang SH, Jeon JY, Lee JH, Kim BS (2010). Heparin-conjugated fibrin as an injectable system for sustained delivery of bone morphogenetic protein-2. Tissue Eng Part A.

[CR52] Zhang L (2010). Glycosaminoglycan (GAG) biosynthesis and GAG-binding proteins. Prog Mol Biol Transl Sci.

[CR53] Zhang H, Migneco F, Lin CY, Hollister SJ (2010). Chemically-conjugated bone morphogenetic protein-2 on three-dimensional polycaprolactone scaffolds stimulates osteogenic activity in bone marrow stromal cells. Tissue Eng Part A.

