# Supplementary material for: Sustained delivery of recombinant human bone morphogenetic protein-2 from perlecan domain I - functionalized electrospun poly (ε-caprolactone) scaffolds for bone regeneration
Source: J Exp Orthop. 2016 Oct 6;3:25. doi: 10.1186/s40634-016-0057-1 (PMC5053971; doi:10.1186/s40634-016-0057-1)
Supplement: Additional file 1: Figure S1. — Perlecan domain I (Dm1) purification and glycosaminoglycan characterization. Perlecan Dm1 purified from HEK293 cells was incubated alone (lanes 2, 9) or with heparitinases 1, 2, 3 and chondroitinase ABC either together (lanes 3, 10) or separately (lanes 5–8). Lane 1 is the molecular weight marker and Lane 4 is all enzymes without Dm1. On the left is a Coomassie stain (lanes 1–8) and on the right is a western blot (lanes 9–10) using a domain I specific antibody (N-20). The arrow head indicates the glycosylated form of Dm I and the arrow indicates the protein core. All enzymes were incubated at 0.1 Units per 10 μg of Dm 1 in a 20 μL of reaction at 37 °C for 4 h. The buffer was 20 mM Tris-HCl, 10 mM NaCl, and 3 mM calcium acetate at pH 8.0. Figure S2. PInDI modified scaffolds controlled rhBMP-2 cumulative release. The absolute amount of rhBMP-2 released from PlnD1-conjugated or unmodified PCL scaffolds over 23 days. (n = 3) Error bars correspond to standard deviation. Figure S3. DNA concentration of W20–17 cells after exposing to rhBMP-2 released from either PlnD1-conjugated or unmodified PCL scaffolds. Fresh W20–17 cultures were used for each time-point (n = 5). (DOCX 227 kb) [file 40634_2016_57_MOESM1_ESM.docx]

**
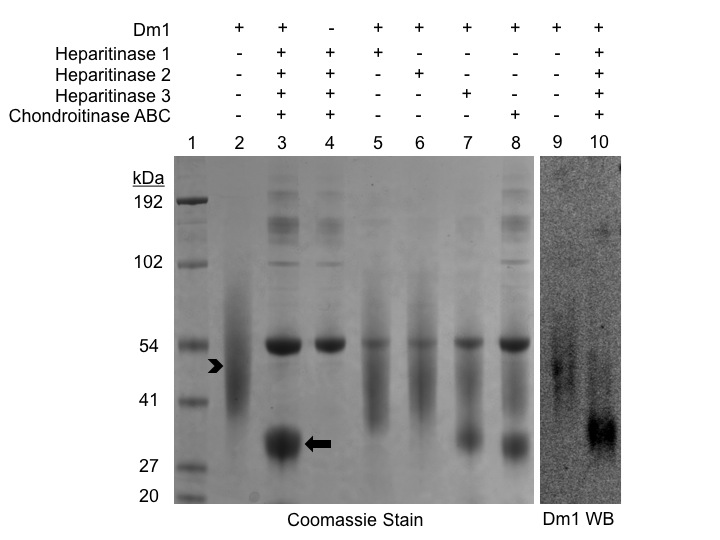
**

Additional file 1: Figure S1: **Perlecan** **domain I (Dm1) purification and glycosaminoglycan characterization**. Perlecan Dm1 purified from HEK293 cells was incubated alone (lanes 2, 9) or with heparitinases 1, 2, 3 and chondroitinase ABC either together (lanes 3, 10) or separately (lanes 5-8). Lane 1 is the molecular weight marker and Lane 4 is all enzymes without Dm1. On the left is a Coomassie stain (lanes 1-8) and on the right is a western blot (lanes 9-10) using a domain I specific antibody (N-20). The arrow head indicates the glycosylated form of Dm I and the arrow indicates the protein core. All enzymes were incubated at 0.1 Units per 10 µg of Dm 1 in a 20 µL of reaction at 37 °C for 4 hours. The buffer was 20 mM Tris-HCl, 10 mM NaCl, and 3 mM calcium acetate at pH 8.0.


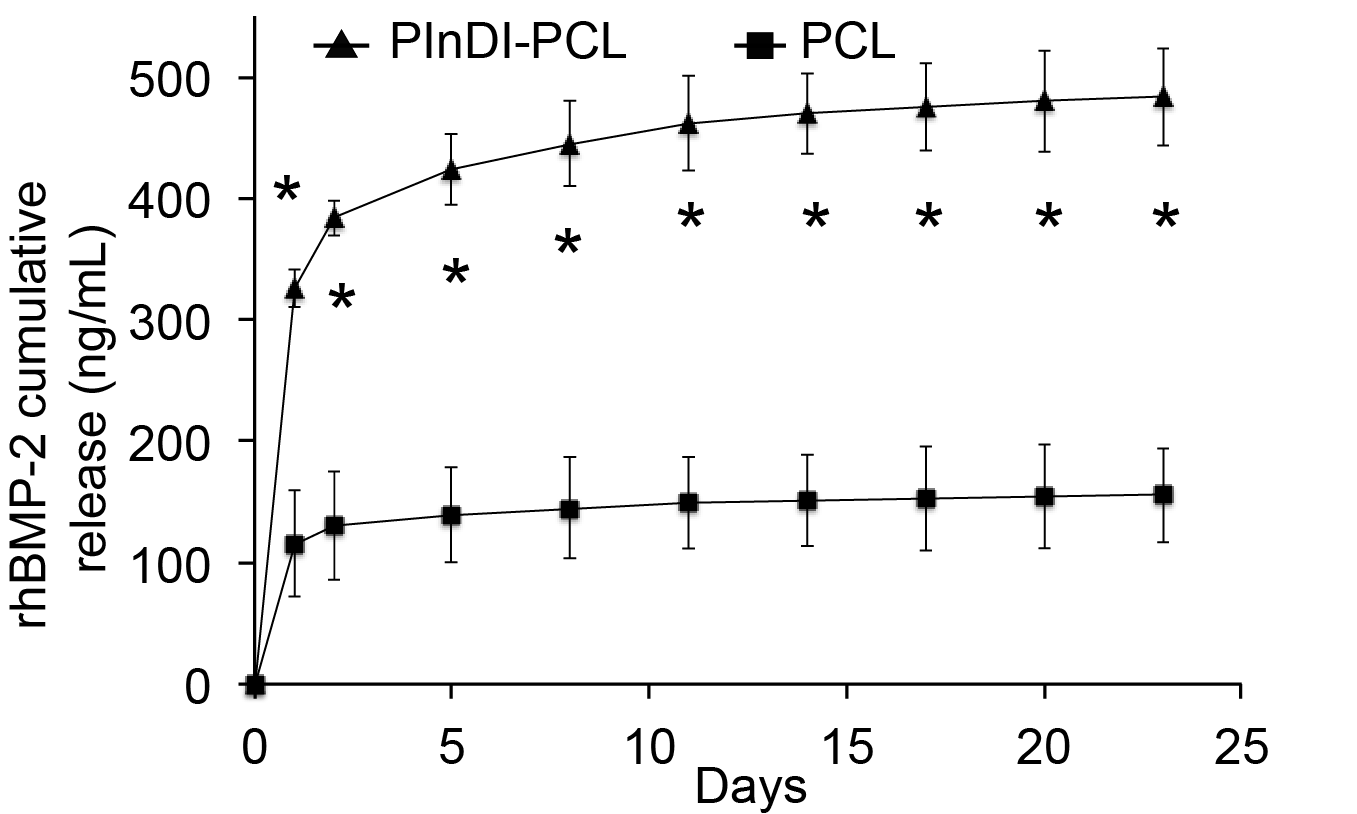


Additional file 1: Figure S2. **PInDI modified scaffolds controlled rhBMP-2 cumulative release.** The absolute amount of rhBMP-2 released from PlnD1-conjugated or unmodified PCL scaffolds over 23 days. (n=3) Error bars correspond to standard deviation.


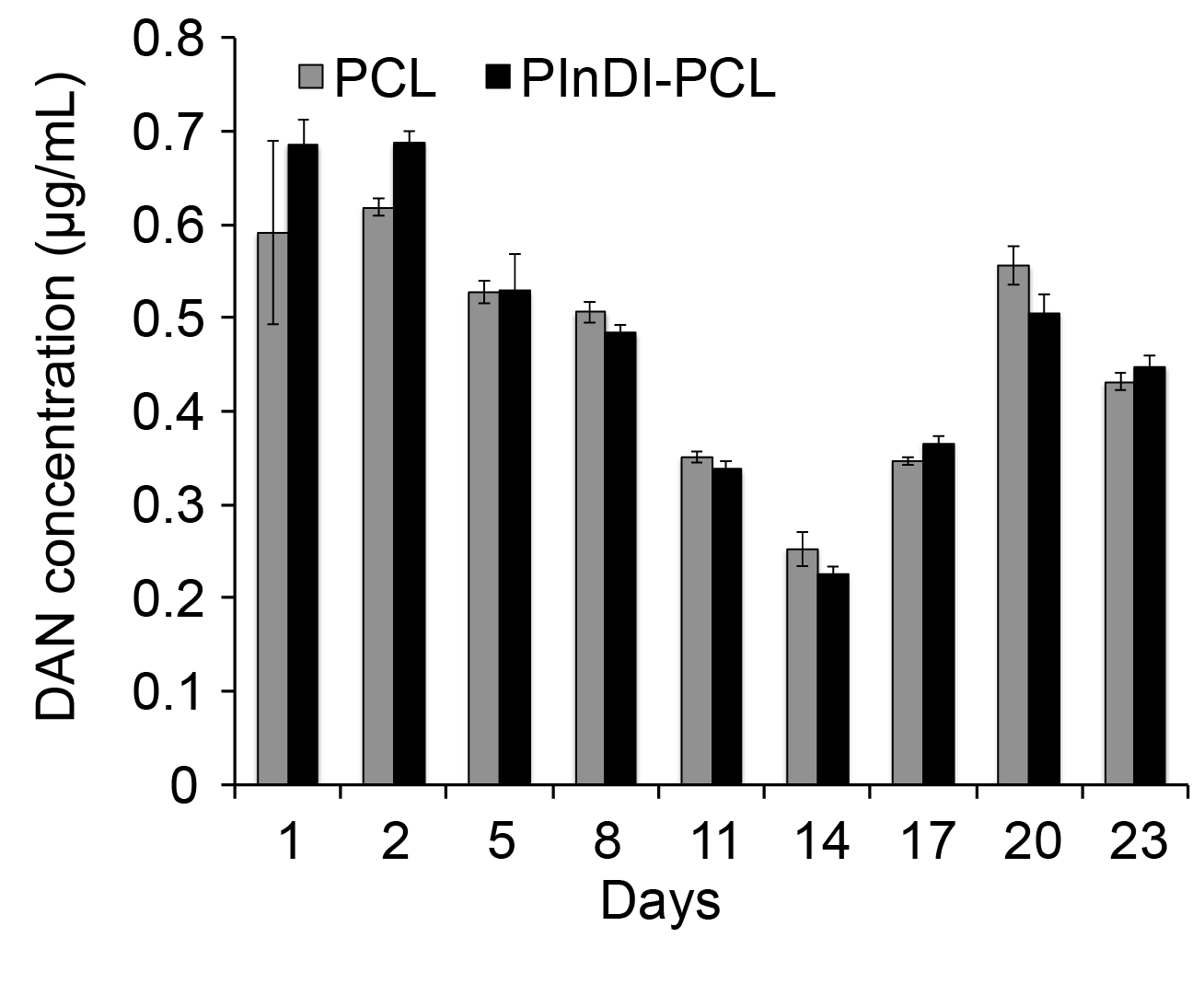


Additional file 1: Figure S3. **DNA concentration of W20-17 cells after exposing to rhBMP-2 released from either PlnD1-conjugated or unmodified PCL scaffolds.** Fresh W20-17 cultures were used for each time-point (n=5).
